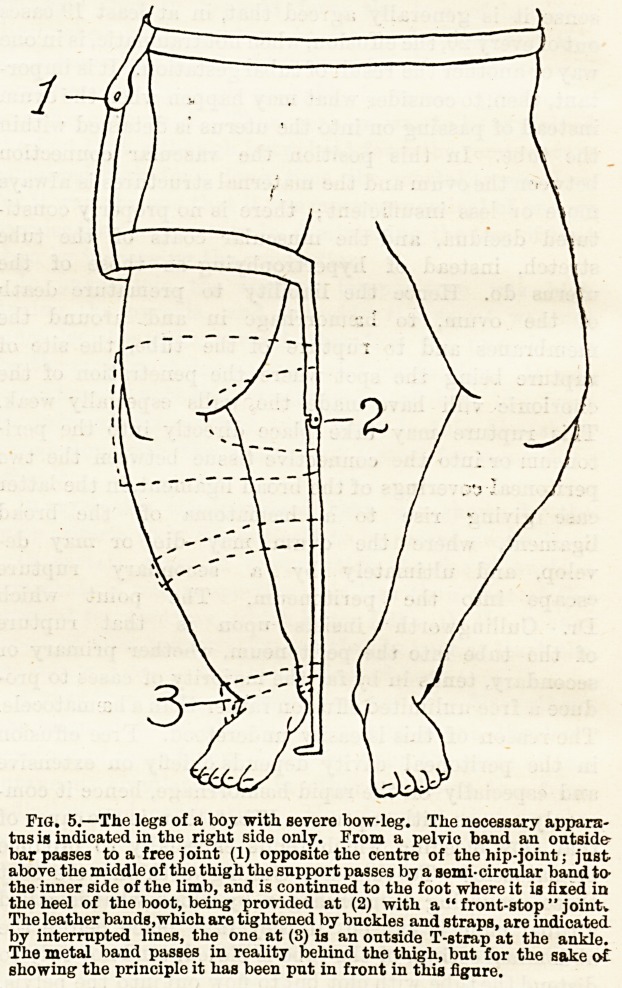# Practical Observations on the Treatment of Common Deformities Caused by Rickets
*An address given at the City Orthopædic Hospital, May 6th, 1897.


**Published:** 1897-06-26

**Authors:** J. Jackson Clarke

**Affiliations:** Assistant-Surgeon at the North-West London and City Orthopædic Hospitals


### PRACTICAL OBSERVATIONS ON THE TREATMENT OF COMMON DEFORMITIES CAUSED BY RICKETS.*

By J. Jackson Clarke, M.B.Lond., F.R.C.S., Assistant-Surgeon at the North-West London and City Orthopædic Hospitals.

(*Concluded from p. 196.*)

**Knock-knee.**—The apparatus for knock-knee of moderate extent is similar to that just described, with the exceptions that the splint is longer, reaching to the pelvis, that it is notched below only, and that instead of the upper band encircling the body, it passes round the posterior half of the body above the buttocks. In knock-knee, the broad central strap passes round the limb above the internal condyle, as shown in Fig. 3.

*An address given at the City Orthopædic Hospital, May 6th, 1897.

Fig. 3.—Chance's splint for knock-knee.

knock-knee is not infrequently accompanied by a slight degree of outward curving of the tibia. When this occurs the two forms of splint may be combined (as shown in Case 2, a little girl aged five).

IG. 4.—A child's back with the moulded splint for rachitic curvature.

**Anterior Tibial Curve.**—A sharp bend at the junction of the middle and lower thirds of the shaft of the tibia is one of the commonest of rachitic deformities, and it is at the same time one of the most difficult to correct by means of splints, owing to the fact that the sharp crest of the tibia lies immediately beneath the skin. In such cases simple wooden splints are not sufficient, and a slightly more complicated appliance is required.

**Ricketty Curves of the Spine.**—The importance of curvature of the spine cannot be over-estimated. These curves form at an earlier age than those of the limbs. The commonest is a C-shaped posterior curve affecting the whole spinal column. An example of this and the

Fig. 5.—The legs of a boy with severe bow-leg. The necessary apparatus is indicated in the right side only. From a pelvic band an outside bar passes to a free joint (1) opposite the centre of the hip-joint; just above the middle of the thigh the support passes by a semi-circular band to the inner side of the limb, and is continued to the foot where it is fixed in the heel of the boot, being provided at (2) with a "front-stop" joint. The leather bands, which are tightened by buckles and straps, are indicated by interrupted lines, the one at (3) is an outside T-strap at the ankle. The metal band passes in reality behind the thigh, but for the sake of showing the principle it has been put in front in this figure.

simple apparatus required for its correction is shown in Case 3, a little boy aged two years. He is wearing a simple moulded and padded leather splint (Fig. 4), which reaches from the nape of the neck to the buttocks, and when the child is sitting the lower end of the splint just rests on the seat. The splint is adjusted by soft straps round the shoulders and a broad band round the abdomen. It thus draws the two ends of the bent spine backwards, and, a most important feature, effects a correction of the deformity without impairing the movements of the ribs. This same apparatus is useful in correcting incipient lateral curvature, which, if left untreated in rickets, is apt to lead to the grossest deformity.

The advantages of the simple apparatus for outward tibial curve, knock-knee, and ricketty spinal curvature may be enumerated thus: (1) They allow free movement, and do not cramp any part of the locomotor or respiratory organs, and so encourage normal development and growth. (2) They are readily understood, and can be adjusted by the mother or nurse. (3) They are inexpensive.

**Deformity of the Chest** due to falling in of the ribs and projection of the sternum (pigeon-breast) cannot be arrested by any mechanical arrangement. The important indications are to remove as far as possible all obstacles which prevent the free entry of air into the lungs; these are chiefly bronchitis and adenoid vegetations. For the former, general hygiene and medical treatment; for the latter, thorough and complete operative measures are required. In all cases of rickets general treatment must be thorough.

Marked deformity in children four years or more of age requires something more in the way of treatment. As an instance this little boy with severe bow-leg may be shown (Fig. 5).

This patient has materially improved since he has worn the apparatus during the last few weeks. For severe genu-valgum an outside iron, with a joint at the hip and an adjustable rack at the knee, and for spinal curvatures Chance's adjustable splints, are required. When the bones have become hard apparatus alone can no longer effect a cure; osteotomy becomes necessary. The object of this paper is to emphasise the importance of early treatment in cases of ricketty deformity, with the view of preventing the need of more complicated apparatus and of operations.

## Figures and Tables

**Fig. 3. f1:**
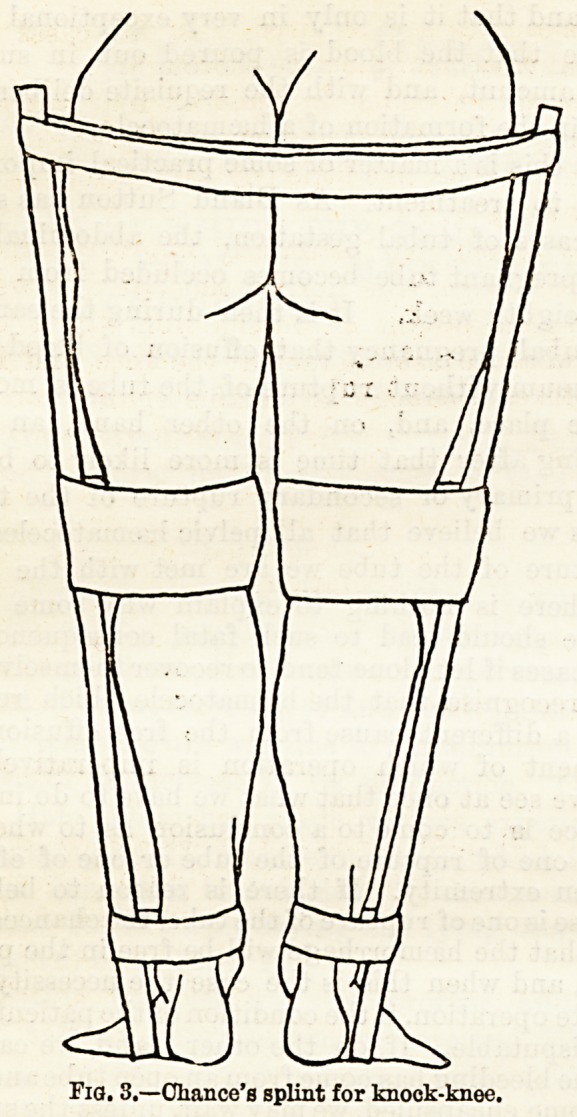


**Fig. 4. f2:**
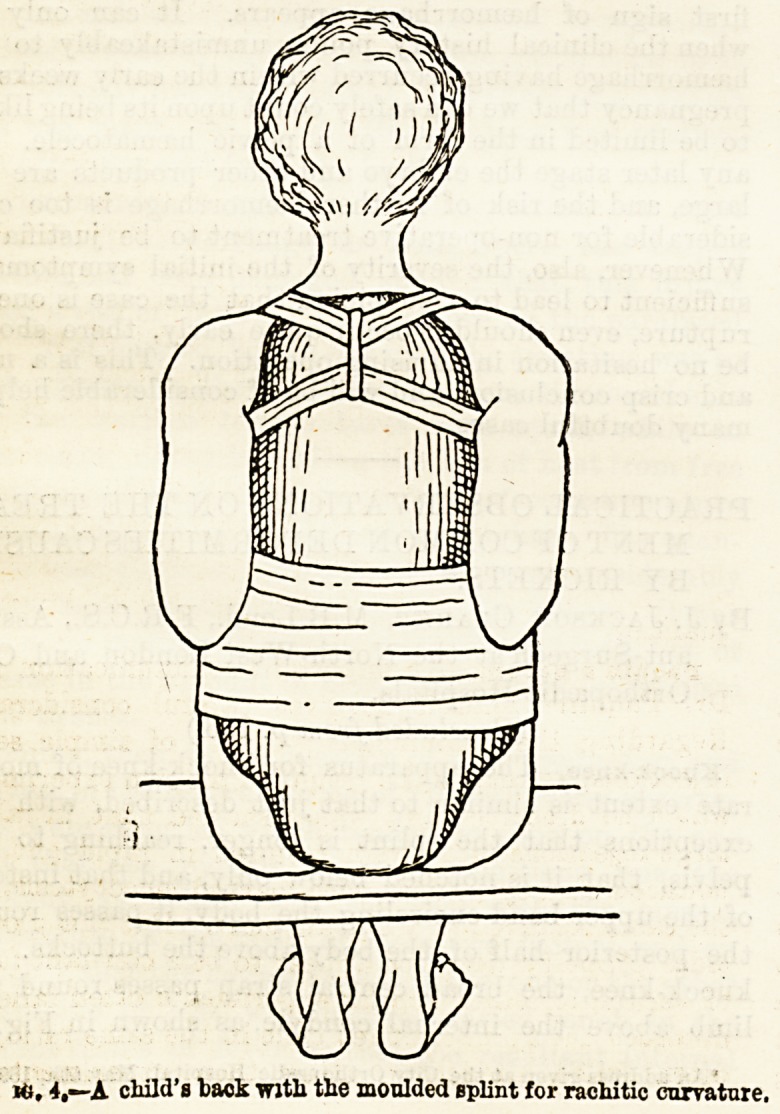


**Fig. 5. f3:**